# Experimentally investigating the structural capacity of slender web tapered built up plate girder with web opening

**DOI:** 10.1038/s41598-025-13111-2

**Published:** 2025-07-31

**Authors:** Mohammed Taher, Ahmed M. Ebid, Sherif M. Ibrahim, Mohamed A. El-Aghoury

**Affiliations:** 1 Faculty of Engineering, Ain Shams University, Cairo, 11535 Egypt; 2 Faculty of Engineering and Technology, Future University in Egypt, Cairo, 11865 Egypt

**Keywords:** Tapered plate girder, Slender web, Web opening, Shear capacity, Civil engineering, Mechanical engineering

## Abstract

Built-up plate girders are widely used in structural applications where hot-rolled beams may not provide sufficient strength or stiffness. To achieve a cost-effective design, tapered plate girders are often employed, allowing for an optimized distribution of material by gradually varying the web depth along the span. In many practical applications, web openings are introduced to accommodate service ducts, utilities, and weight reduction, making them an essential feature in modern steel structures. However, while design codes provide well-established methods for prismatic girders, the web behavior of tapered girders with web openings remains insufficiently investigated, leading to gaps in existing predictive models. This research presents a novel equation to estimate the web capacity of tapered plate girders with web opening, considering parameters such as the panel aspect ratio, web opening size to panel average height ratio and tapering ratio. The equation was validated with the experimental results giving a maximum error of 8%. A comparative study was conducted using different methodologies of steel design standards and previous researches to evaluate their results with the experiments. The results of these methodologies demonstrated significant contradictions. Where some standards underestimated the web capacity by up to 58% while some others overestimated it by up to 45%. The presented formula gives significant improvement in designing the steel plate girders with web opening, hence in structural design. Future research may consider enhancing the equation taking into consideration widening the range of values for taken parameters or by considering more parameters regarding the girders’ geometry, loading and boundary conditions.

## Introduction

Tapered plate girders are broadly used in structural engineering design as a great tool to provide improvement in stress distribution and efficiency in usage of material. Where the depth of such girders varies along their spans according to stress distribution to optimize material usage that leads to reduction in weight and cost. Hence, they are widely used in different structural applications such as bridges and various buildings.

The presence of web openings in girders is necessary for utilities and installments to pass through. This makes it important to consider studying the effect of web opening on shear capacity, bending strength and local buckling of these girders. In addition, the consideration of web tapering will add more complexity in stresses redistribution.

Tapered plate girders with web opening significantly optimize the structural design regarding material usage and costs while maintaining girders’ strength and safety of structures. Therefore, it is important to have comprehensive study on the behavior of such girders. Web openings reduce shear capacity that must be considered significantly in their design. Since current standards and research provide limited guidance, this study is essential for developing more precise provisions for the design of tapered plate girders with web opening.

Recent researchers have proposed studies about tapered plate girders in different cases and conditions to improve the comprehension of their behavior. Trahair et al.^1^ investigated the shear stress distributions in tapered web I-beams. They found that standard beam analysis does not predict shear stress accurately and that modeling normal stress trajectories as radial is more accurate than modeling them as parallel. They highlighted the impact of flange forces and stress gradients on shear distribution. Serror et al.^2^ used finite element analysis to examine the shear strength of tapered web panels. They considered the effect of geometric parameters such as tapering angle, aspect ratio, web slenderness, and transverse stiffeners. They found that the existing design codes, such as AISC and Eurocode 3, do not fully account for the stress redistribution and post-buckling behavior of tapered girders. They proposed modified design formulas to improve shear strength predictions. Tankova et al.^3^ conducted experimental tests on tapered columns, beams, and beam-columns. In addition, they developed a numerical model considering geometric and material imperfections. They provided valuable insights for the performance of tapered girders in different conditions which advanced in the comprehension of how non-uniform structural members behave. Kucukler and Gardner^4^ proposed a stiffness reduction method for evaluating lateral-torsional buckling in web-tapered steel beams that are welded considering the effects of plasticity and imperfections. They validated their method through nonlinear finite element analysis. They improved the predictions of the ultimate strength of such members. Chockalingam et al.^5^ developed a mechanics-based approach to derive an analytical expression for shear stress distribution in tapered I-beams with tapering in webs and flanges. By validating their method through finite element analysis, they provided an accurate and practical tool for designing tapered plate girders considering the complex change in geometry. Ibrahim et al.^6^ investigated the shear strength of slender web-tapered steel members through both experimental and finite element study. Their study considered tapering ratios and web slenderness as parameters. They proposed modification for shear buckling coefficients to improve strength prediction in tapered girders. They also investigated the axial compressive strength of slender webs in tapered steel members using experimental and finite element analysis. They considered web depth and tapering ratio as study parameters. They proposed new calibration for axial buckling coefficients to enhance the prediction of axial strength capacity^7^. S. Ibrahim^8^ investigated in stability analysis of steel frames with tapered members. He derived closed-form equations for bending stiffness and developed design charts for effective buckling length factors for tapered columns. The research demonstrated that existing methods, such as AISC alignment charts, may not capture the behavior of non-prismatic members accurately. Also, it demonstrated that partial tapering of restrained beams significantly reduces the effective buckling length in sway frames while its effect in braced frames remains minimal. Saleh Amin et al.^9^ investigated the effect of web openings on beam-column connections through experimental and numerical studies. Ten full-scale beam-column connections were tested under cyclic loading. They developed numerical model which they validated with the experimental results. The parametric study showed that failure load decreases as openings move closer to the column or get widened. Unreinforced openings had more severe effects than reinforced ones. M. El aghoury et al.^10^ investigated the optimum design of fully composite, unstiffened, built-up hybrid steel girders using three techniques: Generalized Reduced Gradient (GRG), Nonlinear Regression (NLR) and Artificial Neural Networks (ANN). They analyzed structural behavior under different loading conditions and material combinations. They proposed a comparison between the three techniques that highlighted that ANN models achieved higher predictive accuracy in optimizing girder design while reducing material usage and cost. Jagan Jayabalan et al.^11^ examined the buckling load estimation of steel plates with center cut-outs using Artificial Neural Networks (ANN), Gene Expression Programming (GEP), and Evolutionary Polynomial Regression (EPR). They also compared these predictive techniques to evaluate their accuracy in estimating buckling loads under various geometric and material conditions. The results indicated that ANN provides the highest prediction accuracy for structural analysis and optimization. De’nan et al.^12^ conducted a finite element analysis on tapered steel sections with elliptical perforations under shear loading. They demonstrated that optimizing the size and layout of openings could enhance shear buckling capacity and efficiency even with slight weight reductions. R. I. Shahin et al.^13^ introduced an ANN model to predict the elastic critical buckling coefficients of steel beams. They analyzed over 4000 FEA samples. They took into account the tapering ratio, aspect ratio and web slenderness as parameters in their study. The model offers faster and more reliable tool for understanding shear buckling complexity in the design. R. I. Shahin et al.^14^ explored the elastic shear buckling of simply supported girders using finite element analysis. They considered the aspect ratio, tapering ratio and slenderness ratio as their study parameters. As a result, a new equation for the buckling coefficient was proposed that includes a correction factor based on the slender. De’nan et al.^15^ focused on the nonlinear effects of web openings on bending behavior. They revealed that larger openings significantly reduce flexural strength due to stress concentrations.

Despite these advancements, current design codes such as the AISC Steel Construction Manual^16^, AISC 360^17^, AISC Design Guide 02^18^, AISC Design Guide 25^19^, and Eurocode 3^20^ Egyptian Code of Practice for Steel Construction and Bridges (ECP)^21^ provide limited guidance on tapered girders with web openings. These codes mainly address prismatic members and standard conditions. Where they are likely to rely on conservative designs to ensure safety. More detailed guidelines are needed to address tapered girders with web openings, particularly regarding shear capacity, bending strength, and local buckling resistance.

Designing tapered plate girders with web opening comes with unique challenges that current research and design codes don’t fully address. While there have been advancements regarding tapered girders, there’s still a lack of research regarding tapered plate girders that contain web openings, especially in slender webs. This gap calls for further research to develop more precise design guidelines and ensure these structures remain safe and efficient. Therefore, this research aims to experimentally study the tapered plate girders with web opening to increase the understanding of their behavior and to reach a new formula to accurately predict their shear capacities.

The objectives of this research are to explore the structural behavior of tapered, slender plate girders with web opening experimentally. The study focused on web shear capacity and failure mechanism due to web local buckling. The investigation considered the influence of key parameters including the aspect ratio of web panel, the ratio of web opening diameter to the average height of web panel and the tapering ratio. It concerns developing a predictive formula for estimating the web shear capacity of such girders by analyzing the relations between these parameters. In addition, the study evaluates the accuracy of available design codes and methodologies in recent researches against the study results.

## Experimental program

### Materials

**Fig. 1:**
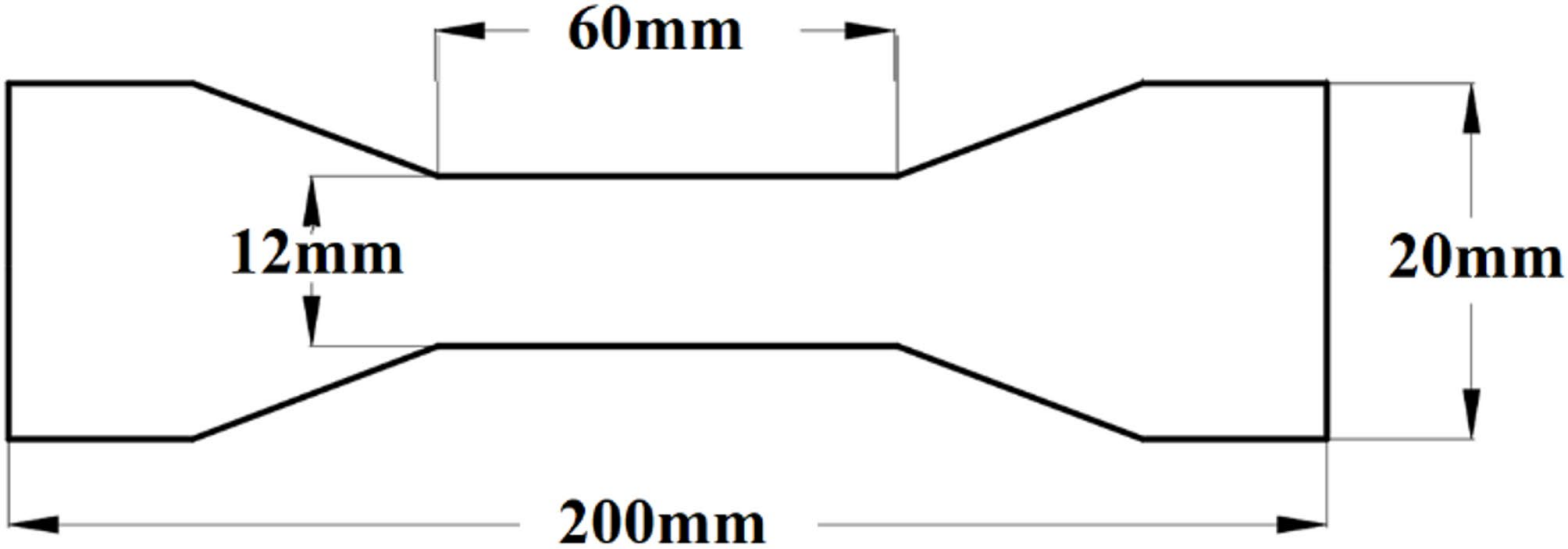
Testing coupons dimensions as per ASTM E8.

**Fig. 2:**
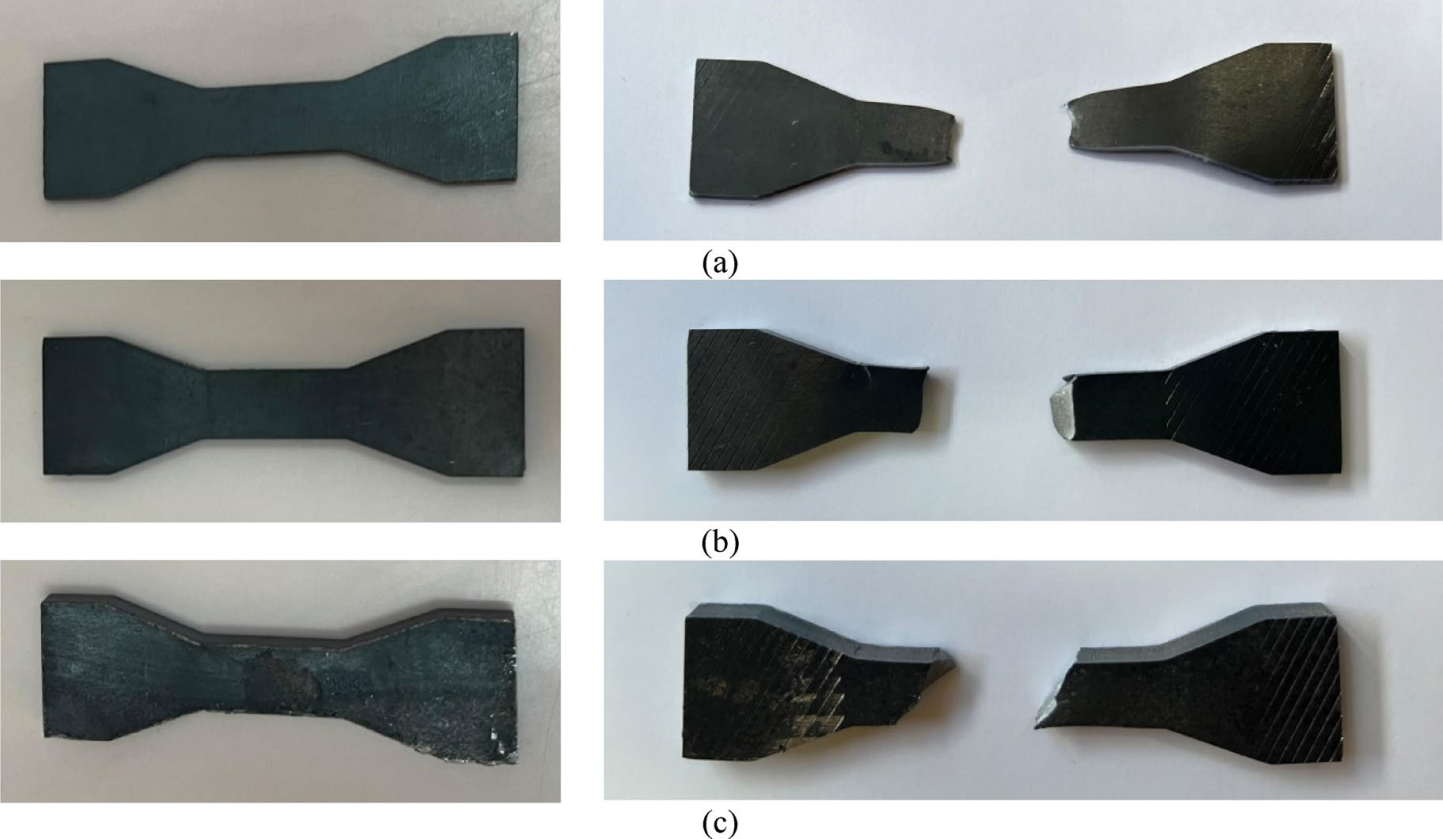
Coupons of girder elements. (**a**) 2 mm Coupon used for web. (**b**) 5 mm Coupon used for stiffeners. (**c**) 8 mm Coupon used for flanges.

**Fig. 3:**
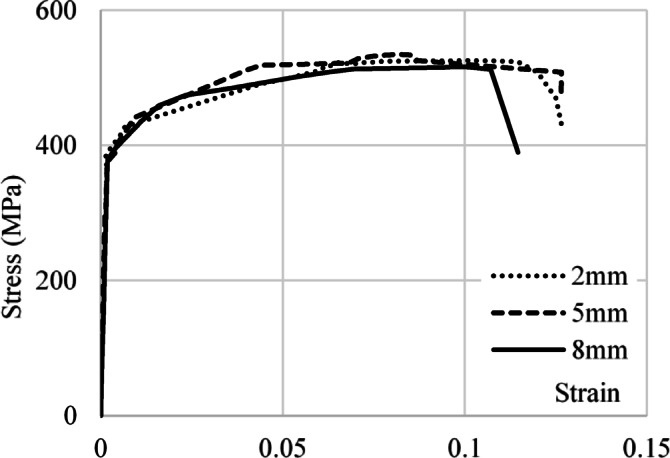
Stress-strain curve of the used materials.

To identify the materials used for each component of the tapered plate girders, small specimens (coupons) were taken from these components and were subjected to tensile tests. The test was performed according to ASTM E8^22^ standards using a universal testing machine (UTM) to determine the properties of materials. Schematic for these coupons dimensions can be shown in Fig. 1 with only the thickness varying according to the girder component where 2 mm thickness is used for web, 5 mm thickness is used for stiffeners and 8 mm thickness is used for flanges. Figure 2 presents the coupons before and after testing. Figure 3 shows the stress-strain curve of the material used. Accordingly, the considered steel properties are Fy = 380 MPa, Fu = 530 MPa, Es = 200 GPA.

### Parameters

**Fig. 4:**
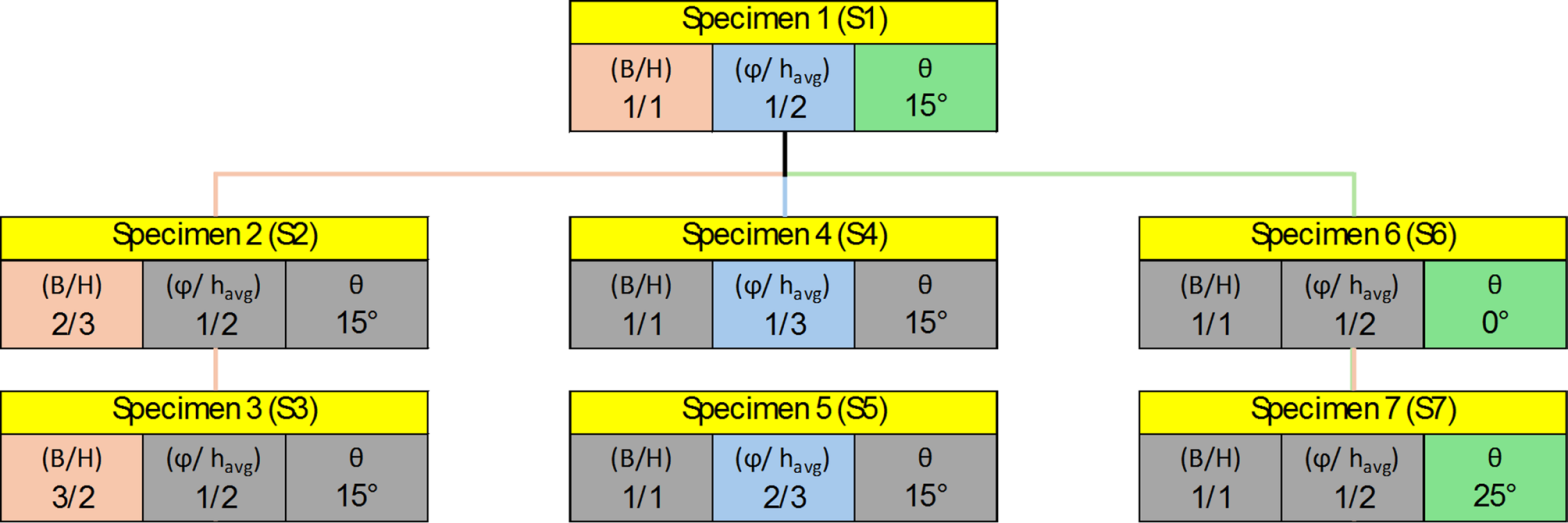
Summary of parameter values for each specimen.

The study examined seven tapered plate girder specimens with web openings, focusing on three key parameters, each varied through different specimens, including a control specimen. Aspect ratio (B/H) is defined as the length of the web panel (B) to the larger height of the girder (H), was varied with values of 2/3, 1.00, and 3/2. The control specimen had an aspect ratio of 1.00. Opening diameter ratio (ϕ/h_avg_.) defined as the ratio of the web opening diameter (ϕ) to the average height (h_avg_) of the girder at the web panel’s mid-height, was varied with values of 1/3, 1/2, and 2/3. The control specimen had an opening diameter ratio (ϕ/h_avg_.) of 1/2. The tapering Ratio (tan θ) indicating the tan angle of inclination of the upper flange, was set at 0°, 0.27°, and 0.47° corresponding to the inclination angles of 0°, 15°, and 25° respectively. Control specimen featured a tapering ratio of 0.27 that corresponds to inclination angle of 15°. Each parameter was studied through different specimens to assess their effects on the performance of the girders, with the control specimen representing the average values for all parameters. Figure 4; Table 1 show the summary of parameter values for each specimen.

### Fabrication

The fabrication of the tapered plate girder specimens was carried out with precision to ensure accurate representation of the design parameters and structural behavior under experimental conditions. The steel plates used for the girder components—webs, flanges, and stiffeners—were cut using a steel laser cutting machine. This method allowed for precise cuts and minimized material wastage, ensuring that each component adhered closely to the specified dimensions.

After cutting, the components were assembled and welded together. All elements of the girders were joined using fillet welds. The welding process was performed with careful attention to detail to avoid any defects that could affect the structural integrity of the specimens. The thickness used for web was 2 mm. All flanges were 100 mm in width & 8 mm in thickness, all stiffeners were 49 mm in width and 5 mm in thickness.

**Fig. 5:**
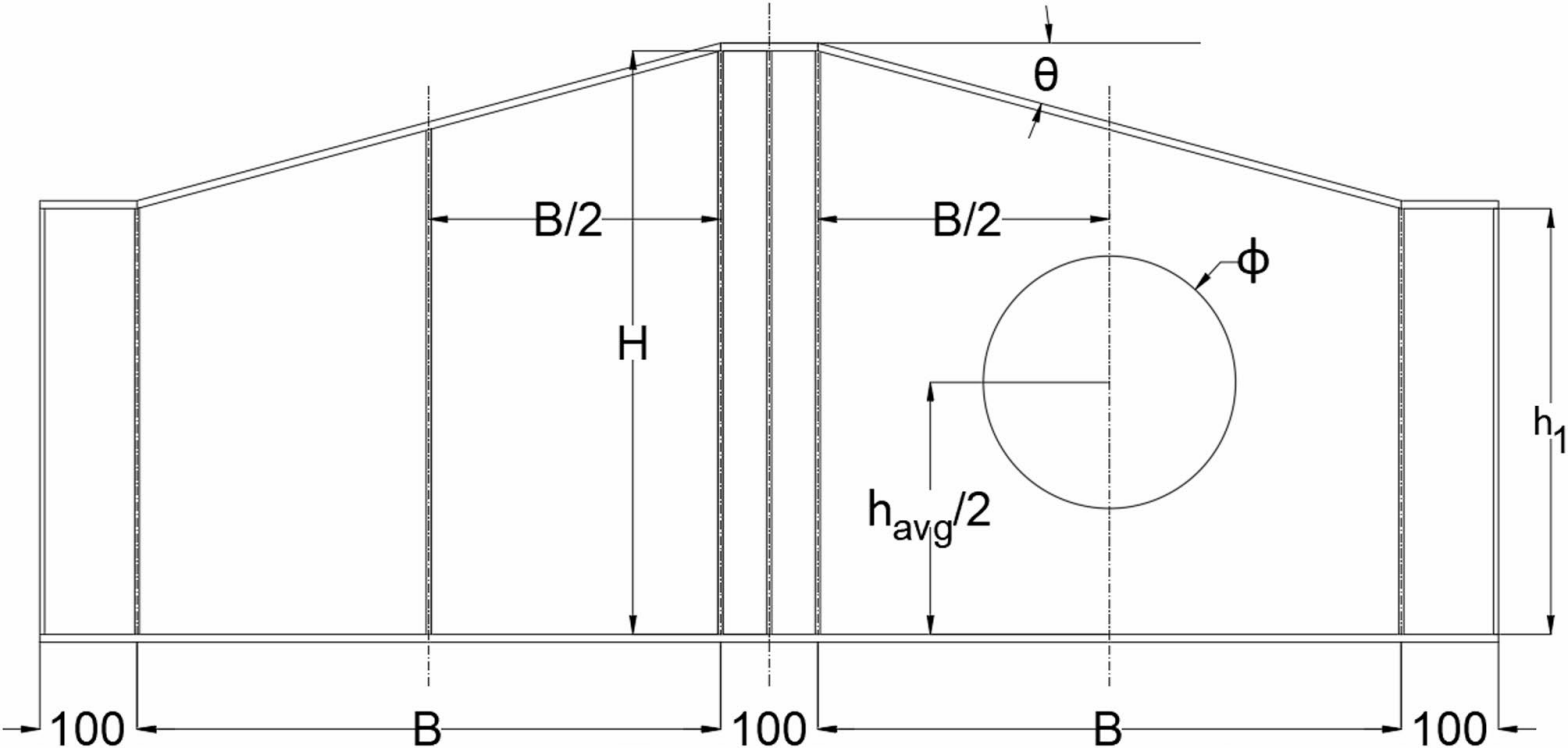
Schematic for specimen dimensions.

Figure 5 shows general schematic of the girder specimens including the dimensions and locations of the web opening. Table 1 shows the detailed dimensions for the specimens where B is the length of the web panel, h is the larger web depth, h_1_ is the smaller web depth, ϕ is diameter of the web opening and θ is the tapering angle of the girder.

**Table 1:** Parameter values and detailed dimensions of specimens.

Specimen ID	B/H	ϕ/h_avg_.	θ	B (mm)	H (mm)	h_1_ (mm)	h_avg_ (mm)	ϕ (mm)	h_avg_./tw
S1	1/1	1/2	15°	600	600	440	520	260	260
S2	2/3	1/2	15°	400	600	550	550	280	275
S3	3/2	1/2	15°	900	600	360	480	240	240
S4	1/1	1/3	15°	600	600	440	520	180	260
S5	1/1	2/3	15°	600	600	440	520	350	260
S6	1/1	1/2	0°	600	600	600	600	300	300
S7	1/1	1/2	25°	600	600	320	460	230	230

### Tolerance

To ensure the accuracy of the experimental setup and to account for any potential imperfections in the girder components, a detailed measurement of the web’s surface was conducted. Imperfections in the web can significantly influence the structural behavior of the girders, particularly in terms of local buckling and stress distribution. Therefore, it was essential to measure and document these imperfections precisely.

**Fig. 6:**
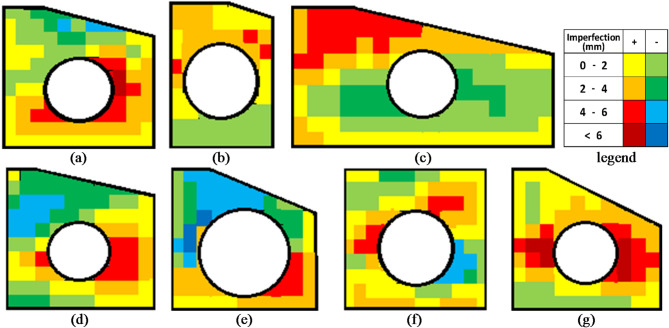
Tolerance graph for each specimen imperfections. (**a**) S1. (**b**) S2. (**c**) S3. (**d**) S4. (**e**) S5. (**f**) S6. (**g**) S7.

A digital vernier caliper with 10 micrometer (10 μm) precision was used to measure deviations in the web surface from a perfect flat plane. A (5 cm × 5 cm) grid was drawn on each girder’s web. At each grid point, surface deviation was recorded. These measurements were used to create contour plots to visually represent the web surfaces imperfections. The contour plot for each specimen is shown in Fig. 6.

### Test setup

The experimental study was conducted with the tapered plate girders configured in a simply supported statical system with a vertical concentrated load at mid-span (3 points bending test). All experiments were conducted in the testing facility of El Shorouk Academy, El Shorouk City, Egypt. The facility provided a 3-points bending testing machine with 1000 kN capacity loading hydraulic jack and 1.0 mm/min. loading rate.

The hinged support was implemented using a steel cylinder that was welded to a base plate, which was securely fastened to the testing frame. This setup allowed the girder to rotate freely about the hinge while preventing any translational movement at that end. On the opposite end, the roller support was represented by a similar steel cylinder that was free to move along the axis of the girder, allowing both rotational freedom and horizontal movement, thus simulating the conditions of a roller support.

**Fig. 7:**
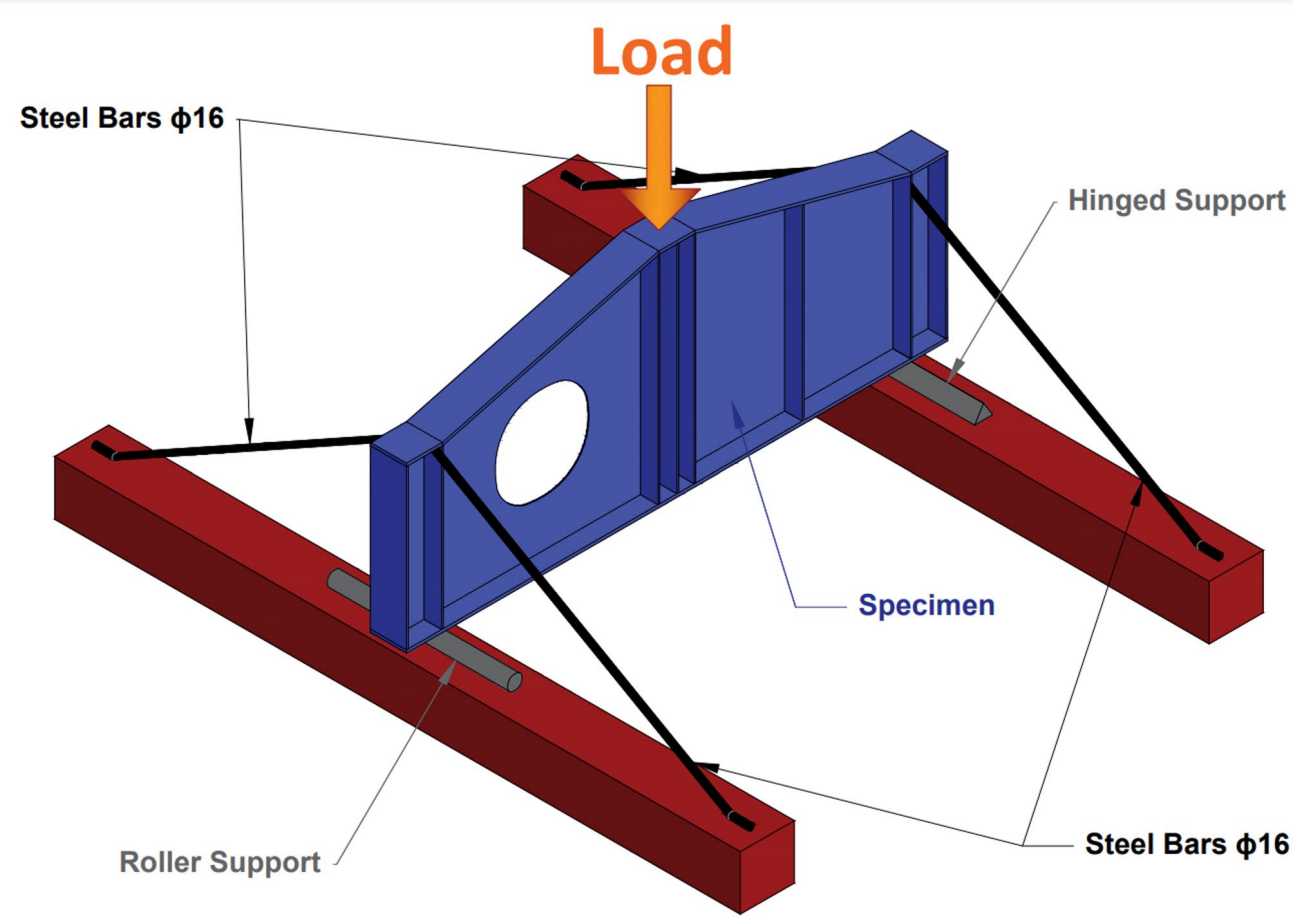
Test setup.

Furthermore, to completely prevent lateral-torsional buckling of girders, steel bars were attached to the top flanges of girders specimens at both ends that were inclined and fixed in the testing frame. In addition, the load application performed at mid-span provided lateral restraint in the upper flange by preventing lateral displacement of the girder during testing. This load application was performed using a hydraulic jack positioned at the mid-span of the girder that was applied vertically. Figure 7 shows details of the test setup, including the positioning of supports, lateral restraints, and the load application.

### Instrumentations

**Fig. 8:**
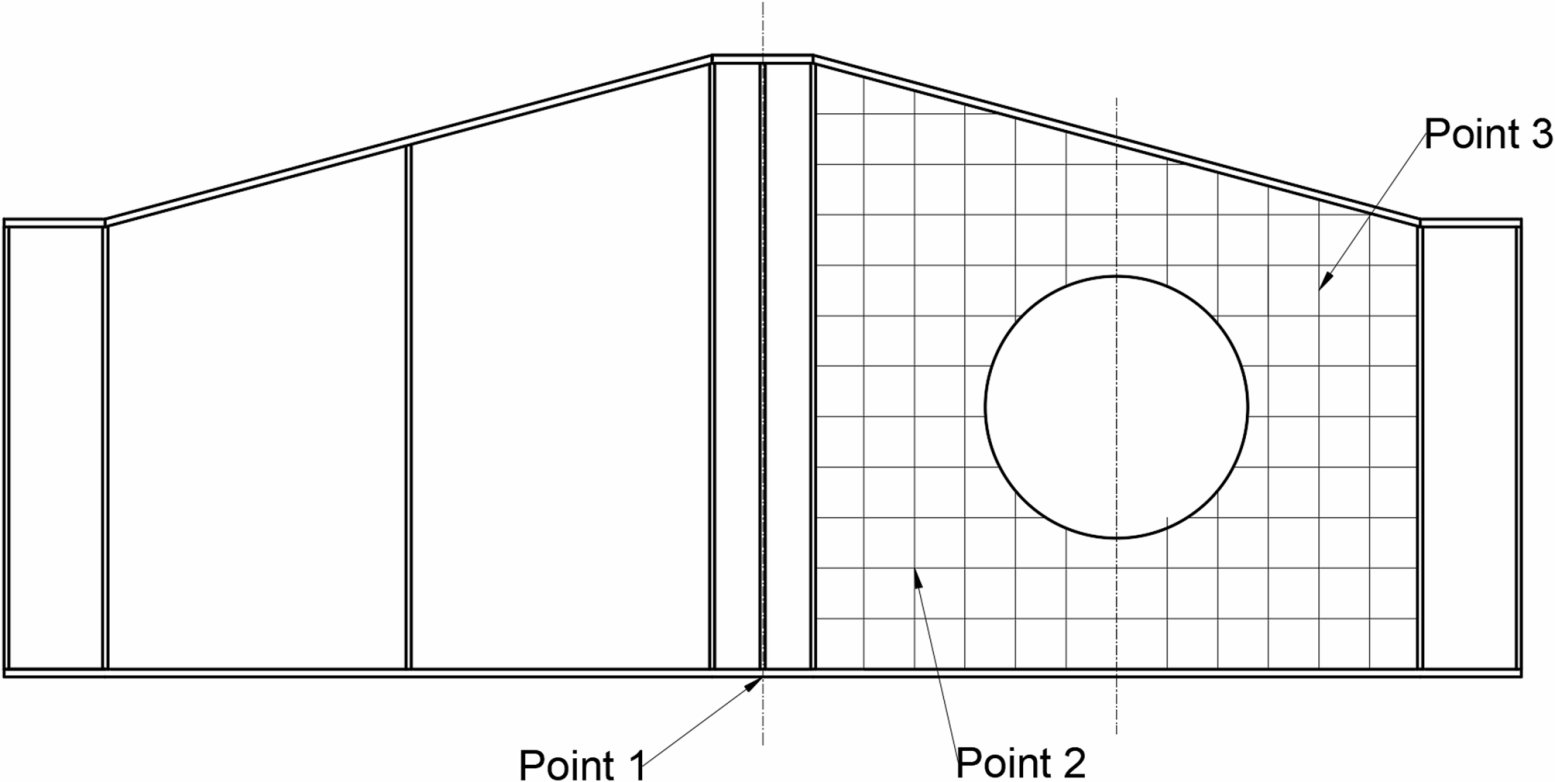
Designated locations for considered points.

**Fig. 9:**
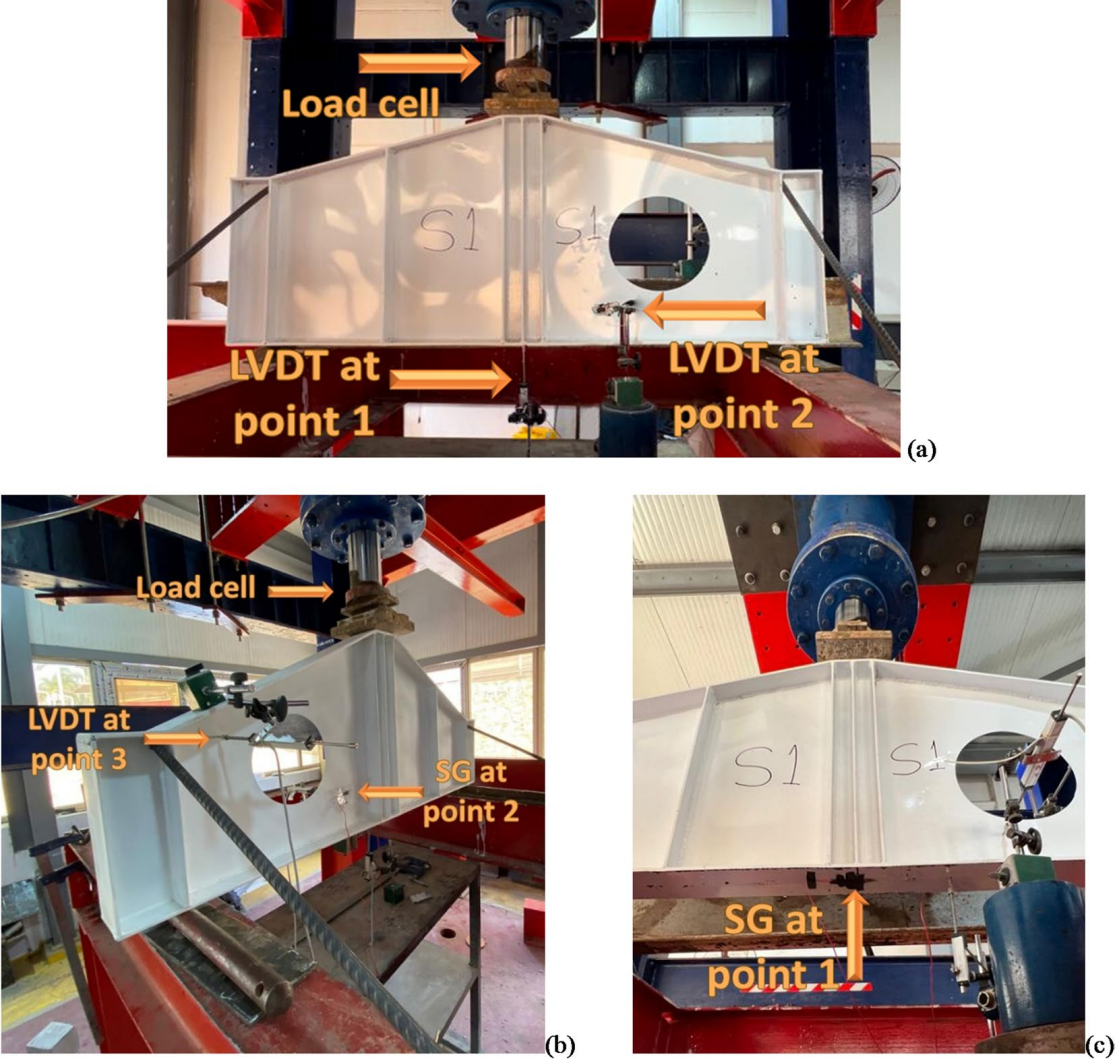
Shots of the instrumentations placements on specimens. (**a**) Front view. (**b**) Back view. (**c**) Bottom view.

Multiple instruments were used in the experimental test to accurately measure and record how the specimens responded. An 800 kN capacity load cell with (0.5%) accuracy was used to apply the vertical load at the mid-span of the girder. Three Linear Variable Differential Transformers (LVDTs) were used to measure vertical deflection and web lateral displacements. One LVDT was positioned at the mid-span of the girder to measure the vertical deflection. And the other two LVDTs were positioned at two points along the diagonal of the web panel to measure lateral displacements. Two strain gauges were used to measure the strains in critical areas of the girders. One strain gauge was attached to the bottom flange at mid-span to measure the longitudinal strain due to bending. The second strain gauge was placed along the diagonal of the web panel with an opening to capture strain variations in this critical region, where stress concentrations were expected. A comprehensive data acquisition system was employed to continuously collect data from all sensors during the tests. The DAQ system ensured synchronized data logging from the load cell, LVDTs, and strain gauges, enabling a detailed analysis of the girders’ response to loading. Figure 8 shows the designated locations for considered points on the web panel. Figure 9 shows shots of the instrumentations’ placements on specimens.

### Testing procedures

The testing procedures were carefully designed to evaluate the structural behavior of tapered plate girders with web openings. Each girder specimen was inspected for defects using the 5 cm x 5 cm grid drawn on the web surface and the digital vernier caliper. The instrumentations of LVDTs and strain gauges were attached at designated locations. Specimens were placed in the test frame, with hinged and roller supports providing simple support conditions. Lateral restraints were installed at both ends using the steel bars and at mid-span using the head jack to prevent lateral-torsional buckling. A vertical load was gradually applied at mid-span using the load cell, with the loading rate controlled to maintain quasi-static conditions (1 mm/min). Data from LVDTs, strain gauges, and the load cell were continuously recorded by the DAQ system. Digital cameras documented deformation and failure modes. The test continued until failure, characterized by significant deformation or fracture, with particular focus on regions around the web openings.

## Results

**Fig. 10:**
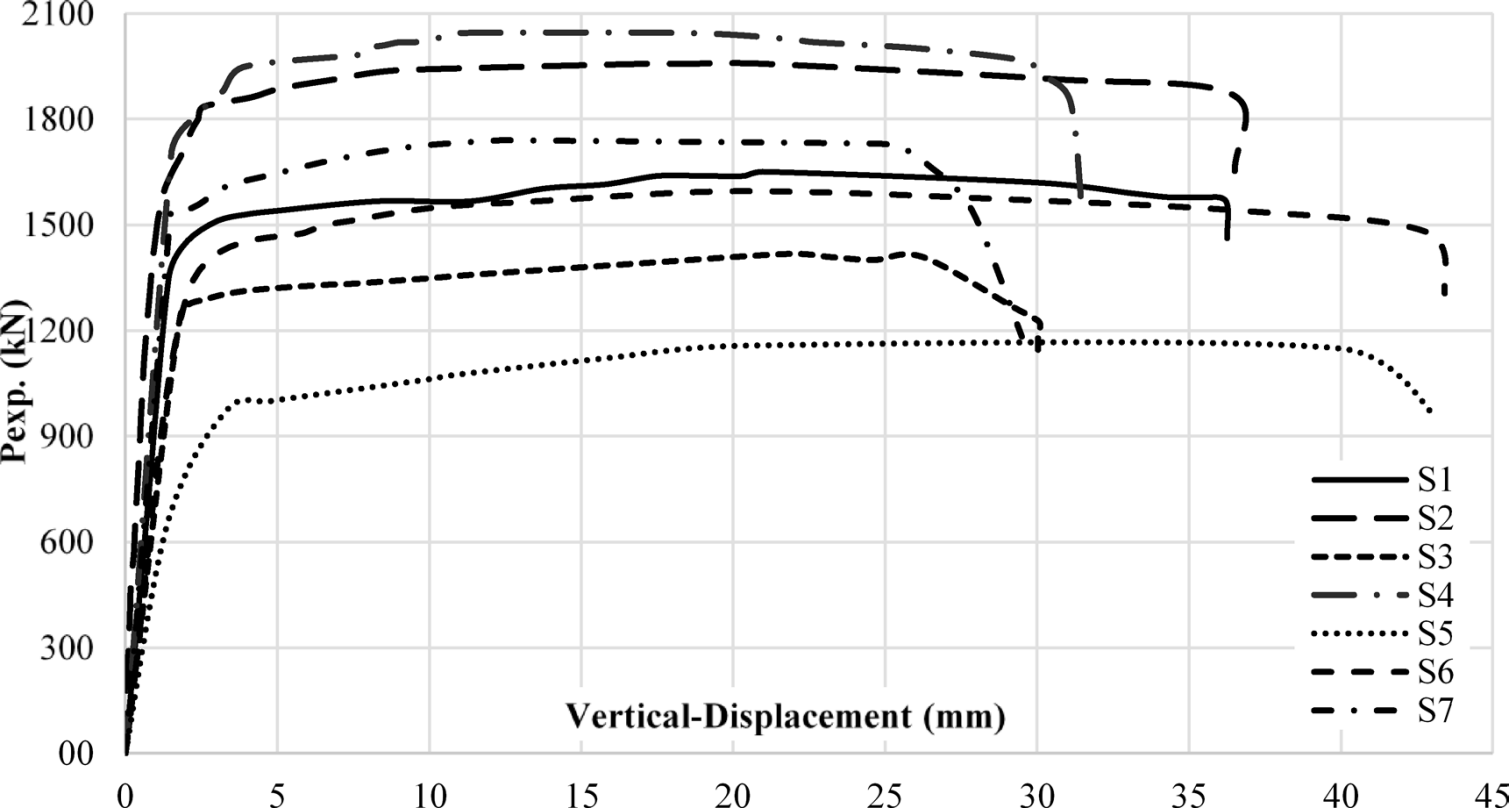
Load-vertical displacement.

**Fig. 11:**
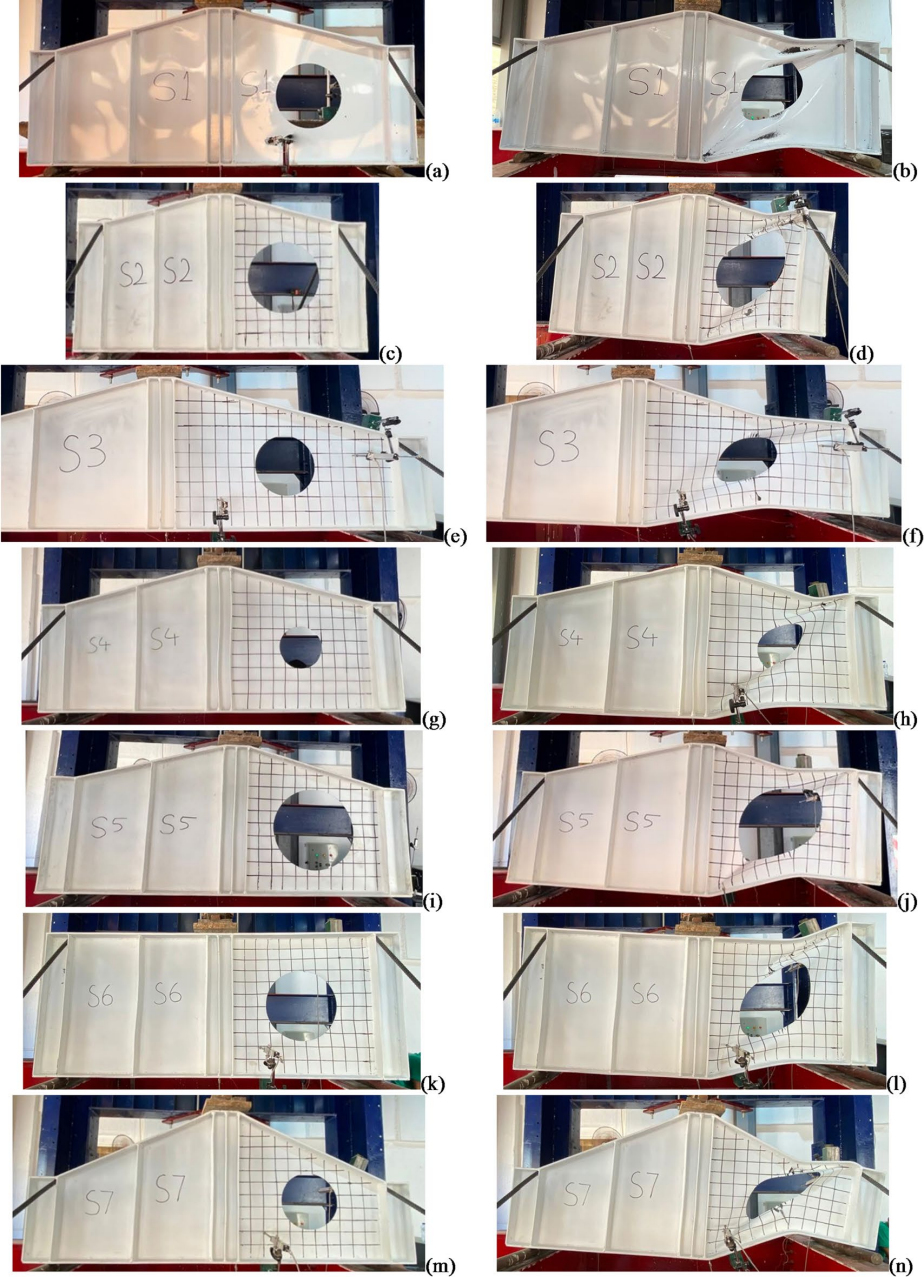
Specimens before and after testing. (**a**) S1 before test. (**b**) S1 after test. (**c**) S2 before test. (**d**) S2 after test. (**e**) S3 before test. (**f**) S3 after test. (**g**) S4 before test. (**h**) S4 after test. (**i**) S5 before test. (**j**) S5 after test. (**k**) S6 before test. (**l**) S6 after test. (**m**) S7 before test. (**n**) S7 after test.

Data was collected for buckling load, ultimate load, deflections, and strain values for all specimens. The results showed varying degrees of stress concentrations around the openings, which generally led to reduced shear capacity depending on the considered key parameters. Table 2 shows the values measured from the attached instrumentations on each specimen. Initiation of local buckling was determined using strain data collected from strain gauges and was confirmed by observation. Figure 10 shows the load to vertical displacement at mid-span for each specimen. Figure 11 shows shots of specimens before and after test.

**Table 2:** Values measured from the attached instrumentations.

	Item	Unit	Specimen						
			S1	S2	S3	S4	S5	S6	S7
Buckling	P_b_.	(kN)	141.0	164.0	101.0	192.0	102.0	131.0	150.0
	δ1_b_.	(mm)	1.8	2.1	1.9	2.2	3.3	1.9	1.7
	δ2_b_.	(mm)	0.2	− 2.8	6.6	− 5.5	− 12.3	6.6	8.8
	δ3_b_.	(mm)	3.4	− 6.4	2.4	− 6.7	15.0	− 8.5	− 3.0
	$\epsilon$1_b_.	(µm/mm)	290	490	391	760	373	321	382
	$\epsilon$2_b_.	(µm/mm)	1920	− 1820	554	− 1849	− 1734	1784	1752
Ultimate	P_ult_.	(kN)	167.00	195.75	141.81	204.45	115.71	159.20	174.87
	δ1_ult_.	(mm)	21.3	21.0	21.8	18.5	20.6	22.6	12.4
	δ2_ult_.	(mm)	18.0	− 17.2	16.5	− 15.2	− 50.0	17.8	23.5
	δ3_ult_.	(mm)	21.2	− 15.5	27.5	− 13.5	87.7	− 17.4	− 21.5
	$\epsilon$1_ult_.	(µm/mm)	190	259	568	839	157	313	544
	$\epsilon$2_ult_.	(µm/mm)	2784	− 9152	6046	− 2131	− 8157	6932	7852

where P_b_.: Buckling load. δ_1b_. : Vertical displacement at point 1 at buckling load. δ_2b_. : Lateral displacement at point 2 at buckling load. δ_3b_. : Lateral displacement at point 3 at buckling load. $\epsilon$_1b_. : Strain gauge at point 1 at buckling load. $\epsilon$_2b_. : Strain gauge at point 2 at buckling load. P_ult_. : Ultimate load. δ_1ult_. : Vertical displacement at point 1 at ultimate load. δ_2ult_. : Lateral displacement at point 2 at ultimate load. δ_3ult_. : Lateral displacement at point 3 at ultimate load. $\epsilon$_1ult_. : strain gauge at point 1 at ultimate load. $\epsilon$_2ult_. : strain gauge at point 2 at ultimate load.

## Discussion

### Aspect ratio

The aspect ratio, defined as the ratio of the length of the web panel (B) to the larger height of the girder (), significantly influences the shear behavior and buckling resistance of tapered plate girders with web openings. In this study, it was observed that increasing the aspect ratio, while keeping the height and thickness constant, increased the panel area. This larger panel area resulted in greater slenderness, making the web more susceptible to shear buckling due to the increased unsupported surface area where shear deformations could develop.

**Fig. 12:**
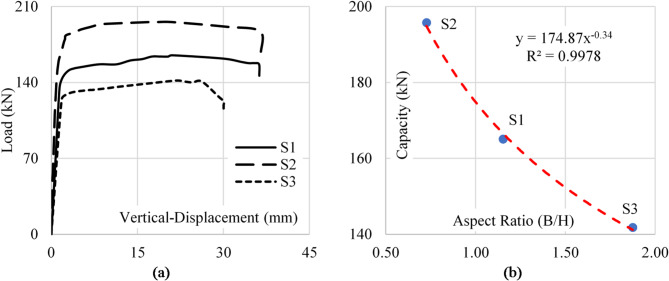
Aspect ratio impact on sample capacity. (**a**) Load–disp. (**b**) Capacity–aspect ratio.

Specimens with lower aspect ratios (e.g., B/h = 2/3) demonstrated better buckling resistance as the reduced panel area limited shear deformations and stress intensification. Conversely, specimens with higher aspect ratios (e.g., B/h = 3/2) showed reduced shear capacity and greater vulnerability to local buckling, emphasizing the critical role of the web panel’s dimensions in shear performance. Figure 12 shows the load-vertical displacement curves for the considered specimens.

### Diameter/average height (ϕ/h_avg_.)

The parameter (ϕ/h_avg_.) (the ratio of the web opening diameter to the average height of the girder) plays a crucial role in determining the shear strength of tapered plate girders with web openings. As the web opening diameter (ϕ) increases, the effective height of the web resisting shear forces decreases linearly. Consequently, the area available to resist shear stresses also decreases linearly, directly impacting the girder’s shear capacity.

**Fig. 13:**
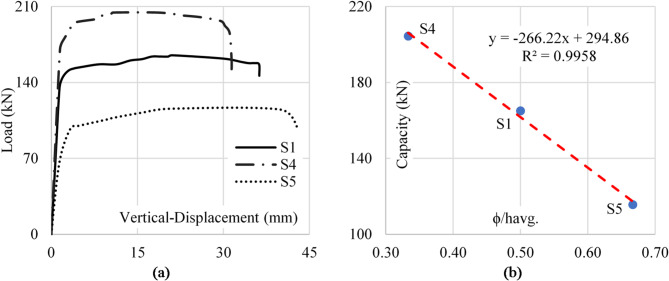
(Diameter/average height) impact on sample capacity (**a**) Load–disp. (**b**) Capacity–diameter/average height.

In the experimental results, specimens with larger (ϕ/h_avg_ ) values exhibited a notable reduction in shear resistance due to the diminished web area available for stress distribution. The reduction in web height effectively increased stress concentration around the openings, which further exacerbated local buckling and early shear failure. Conversely, smaller web openings preserved more of the shear-resisting area, leading to improved structural performance. Figure 13 shows the load-vertical displacement curves for the considered specimens.

### Tapering ratio

The tapering ratio, defined as the tangent of the upper flange inclination angle, impacts the shear capacity of tapered plate girders with web openings. Experimental results indicated that as the tapering ratio increased, the shear capacity of the girders improved. This positive correlation can be attributed to the enhanced web depth in tapered sections, which decreases the slenderness in the tapered area making it more resistant to buckle.

**Fig. 14:**
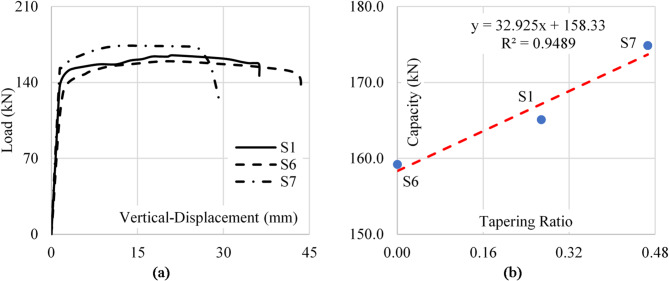
Tapering ratio impact on sample capacity (**a**) Load–disp. (**b**) Capacity–tapering ratio.

Specimens with lower tapering ratios demonstrated a lower response to applied shear loads due to the increased slenderness along the web panel. Figure 14 shows the load-vertical displacement curves for the considered specimens.

### Development of the shear capacity prediction equation

A novel equation was developed to predict the shear capacity (V_shear_) of tapered plate girders with web opening. The equation considers the combined influence of three critical parameters: the aspect ratio (B/H), the web opening size relative to the average height (D/h_avg_), and the tapering ratio (tanθ). The general form of the equation is expressed is Eq. 1.

The shear strength of tapered, slender plate girder with web opening is considered as follows:

1 $$\:{V}_{shear}={V}_{max\:}\cdot \:\:{\eta\:}_{1}\:\cdot \:\:{\eta\:}_{2}\cdot \:\:{\eta\:}_{3}\:\cdot \:\:{\eta\:}_{4}=(0.6\:\cdot \:\:{f}_{y}\:\cdot \:\:{A}_{w})\:\cdot \:\:{\eta\:}_{1}\:\cdot \:\:{\eta\:}_{2}\cdot \:\:{\eta\:}_{3}\:\cdot \:\:{\eta\:}_{4}$$

where *f*_*y*_ is the yield stress of steel, (0.6. *f*_*y*_*)* is the shear strength of steel. *A*_*w*_ is the gross area of cross-sectional area of web at the average height of the girder (h_avg_. tw). *V*_*max*_ is the theoretical shear capacity of the web without considering the opining and the slenderness. *η*_*1*_ is a correction coefficient for opening ratio. *η*_*2*_ is a correction coefficient for web slenderness. *η*_*3*_ is a correction coefficient for aspect ratio. *η*_*4*_ is a correction coefficient for tapering ratio.

*η*_*1*_ is considered as the ratio between the net cross-sectional area of web with opening and the net cross-sectional area of web without opening ($\:{A}_{{W}_{net}}/{A}_{w}$) = (h_avg_ - φ) / h_avg_ as (t_w_) is constant.

For the considered slenderness ratio range (230 < h_avg_./t_w_ < 300) as shown in Table 1, the relation between the Euler buckling stress and slenderness ratio could be considered linear. Accordingly the web capacity is linearly proportional with the inverse of slenderness ration, and then, *η*_*2*_ = (t_w_ / h_avg_).

Therefore, the shear strength of tapered plate girder with web opening considering the web opening diameter and its slenderness ratio is calculated as shown in Eq. 2.

2 $$\:{V}_{shear}=0.6\:\cdot \:\:{f}_{y}\:\cdot \:\:{A}_{{w}_{net}}\:\cdot \:\:\frac{{t}_{w}}{{h}_{avg.}}\:\cdot \:\:{\eta\:}_{3}\:\cdot \:\:{\eta\:}_{4}$$

where: $$\:{A}_{{w}_{net}}=\:{t}_{w\:}\times\:\:({h}_{avg.}-\:{\upvarphi\:})$$

**Fig. 15:**
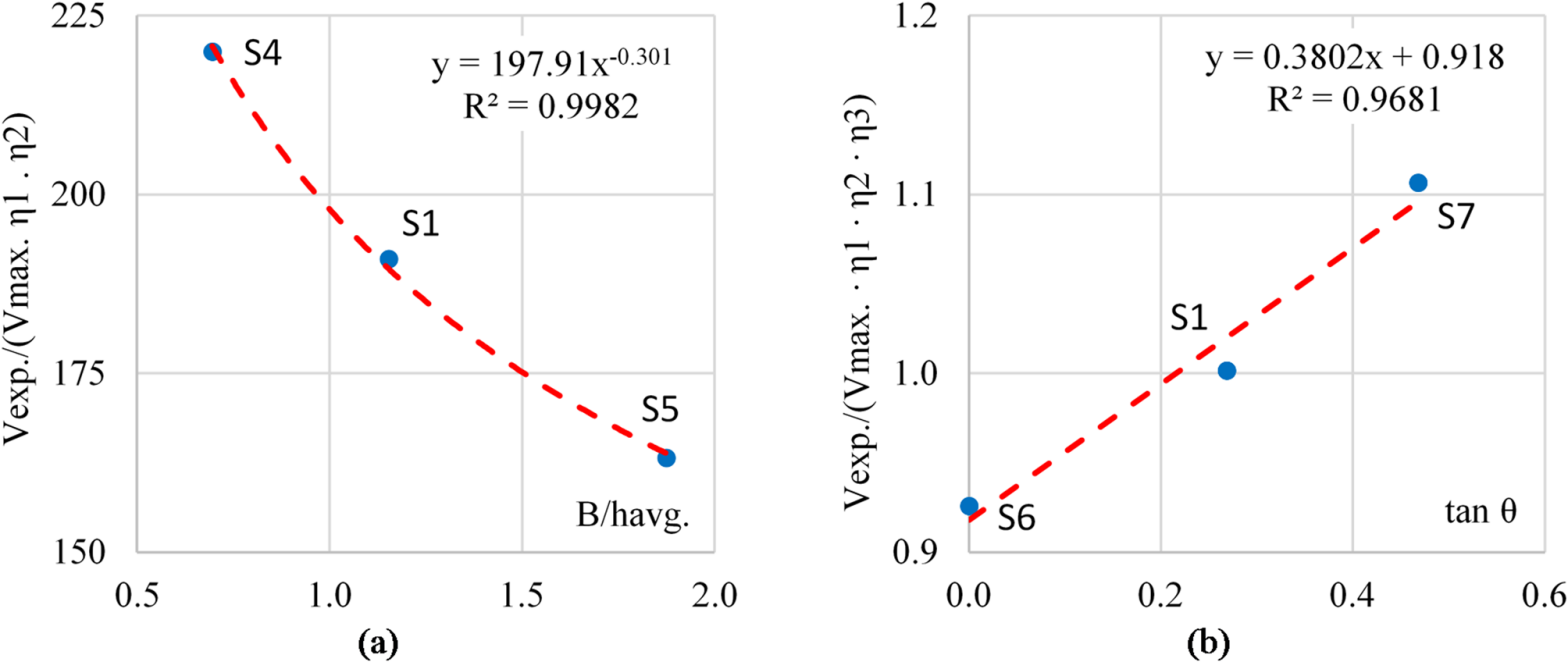
(**a**) Aspect ratio coefficient (η3), (**b**) Tapering ratio coefficient (η4).

To determine the values of η3, the relation between the experimental shear capacity (V_exp_) and (V_max_. *η*_*1*_ *. η*_*2*_) were plotted for three samples with different aspect rations (B/h_avg_) but the same tapering ratio (tan θ) to eliminate the effect of *η*_*4*_. These samples are S1, 4, 5. Figure 15-a shows the formula of the best fitting curve for these three samples. Accordingly, η_3_ ≈ 200 (B / h_avg_) ^−0.3^. Therefore, the shear capacity of such plate girders considering the aspect ratio can be calculated using Eq. 3.

3 $$\:{V}_{shear}=0.6\:\cdot \:\:{f}_{y}\:\cdot \:\:{A}_{{w}_{net}}\:\cdot \:\:\frac{{t}_{w}}{{h}_{avg.}}\:\cdot \:\:200\:\cdot \:\:\sqrt[3]{\frac{{h}_{avg.}}{B}}\:\cdot \:\:{\eta\:}_{4}$$

Finally, to determine the *η*_*4*_ the relation between the experimental shear capacity (V_exp_) and (V_max_. *η*_*1*_ *. η*_*2*_ *. η*_*3*_) were plotted for three samples with different tapering rations (tan θ). These samples are S1, 6, 7. Figure 15b shows the formula of the best fitting curve for these three samples. Accordingly, *η*_*4*_ = 0.38 tan(θ) + 0.92. Therefore, the shear capacity of plate girders considering all the mention parameters is calculated using Eq. 4.

4 $$\:{V}_{shear}=0.6\:\cdot \:{f}_{y}\:\cdot \:{A}_{{w}_{net}}\:\cdot \:\:200\:\cdot \:\:\frac{{t}_{w}}{{h}_{avg.}}\:\cdot \:\:\sqrt[3]{\frac{{h}_{avg.}}{B}}\:\left(0.38\text{tan}\theta\:+0.92\right)$$

Table 3 shows the comparison between the shear measured in the experimental test and the calculated shear capacity from the proposed equation for each specimen, where (Vcalc.) is the calculated shear load from Eq. 4, and (Vexp.) The measured load from experimental test.

**Table 3:** Comparison between the shear measured in the experimental test and the calculated shear capacity from the proposed equation.

Specimen	t_w_	h_avg_.	ϕ	A_w_net_	B	θ	tan θ	V_calc_.	V_exp_.	V_calc_./V_exp_.
ID	cm	cm	cm	cm^2^	cm	Degree	–	kK	kN	–
S1	0.2	52	26	5.2	60	15	0.27	83.40	82.50	101%
S2	0.2	55	28	5.4	40	15	0.27	95.50	97.50	98%
S3	0.2	48	24	4.8	90	15	0.27	70.90	70.50	101%
S4	0.2	52	18	6.8	60	15	0.27	109.10	102.00	107%
S5	0.2	52	35	3.4	60	15	0.27	54.50	57.50	95%
S6	0.2	60	30	6	60	0	0.00	77.80	80.00	97%
S7	0.2	46	23	4.6	60	25	0.47	86.70	87.50	99%

This formula is limited to the range of study considered in this research where web slenderness ratio is ranged between 230 and 300, aspect ratio of web panel is from 2/3 to 3/2, the diameter ratio is from 1/3 to 2/3 and the tapering angle from 0° (no tapering) to 25°.

## Comparison with earlier studies and design codes

### AISC 360

AISC 360 specifications primarily address prismatic members, with shear strength predictions based on section-by-section evaluations. However, applying these methods on the girders with opening in the web panel have notable limitations, including the lack of specific provisions for tapered members and a simplified approach to web buckling analysis. The procedures often underestimate the capacity of unstiffened tapered members by ignoring the strength contributions of geometric tapering, leading to overly conservative results by an average of 58% conservation. Which also adds up with the findings of Ibrahim et al.^6,7^ that tapering of plate girders has a positive effect on their capacities. Therefore, it can be noted that all of the code’s predictions of specimens’ shear capacities are conservative. Equations from Eq. 5 to Eq. 7 show the procedure to determine the shear capacity using this code.

5 $$\:{V}_{n}=0.6{F}_{y}{A}_{w}{C}_{v}$$

where F_y_ is the steel yield stress, A_w_ is the web cross sectional area and C_v_ is the shear buckling stress to shear stress ratio. And can be calculated using Eq. 6 to 8. Mentioning that opening in web is taken into consideration by substituting A_net_ instead of A_w_, where A_net_ is the web cross sectional reduced by the web opening cross sectional area

6 $$\:{C}_{v}=1.0\:if\:\frac{h}{{t}_{w}}<1.10\sqrt{{k}_{v}E/{F}_{y}}$$

7 $$\:{C}_{v}=\frac{1.10\sqrt{{k}_{v}E/{F}_{y}}}{h/{t}_{w}}\:if\:1.10\sqrt{{k}_{v}E/{F}_{y}\:}<\:h/{t}_{w\:}<\:1.37\sqrt{{k}_{v}E/{F}_{y}\:}$$

8 $$\:{C}_{v}=\frac{1.51{k}_{v}E}{{F}_{y}{\left(h/{t}_{w}\right)}^{2}}\:if\:1.10\sqrt{{k}_{v}E/{F}_{y}\:}<\:h/{t}_{w\:}<\:1.37\sqrt{{k}_{v}E/{F}_{y}\:}$$

where h and t_w_ are the web depth and thickness respectively, E is the steel modulus of elasticity and k_v_ is the shear buckling coefficient of simply supported prismatic web that can be determined by Eqs. 9 & 10.

9 $$\:{k_{v}\:=\:5\:\left(\text{f}\text{o}\text{r}\:\text{w}\text{e}\text{b}\:\text{p}\text{a}\text{n}\text{e}\text{l}\text{s}\:\text{w}\text{i}\text{t}\text{h}\text{o}\text{u}\text{t}\:\text{s}\text{t}\text{i}\text{f}\text{f}\text{e}\text{n}\text{e}\text{r}\text{s}\right)}_{}$$

10 $$\:{k}_{v}=5+\frac{5}{{\left(a/h\right)}^{2}}\left(\text{f}\text{o}\text{r}\:\text{w}\text{e}\text{b}\:\text{p}\text{a}\text{n}\text{e}\text{l}\text{s}\:\text{w}\text{i}\text{t}\text{h}\:\text{s}\text{t}\text{i}\text{f}\text{f}\text{e}\text{n}\text{e}\text{r}\text{s}\right)$$

where α is distance between transverse stiffeners. h is the distance between flanges.

### AISC design guide 2

AISC Design Guide 2 provides practical recommendations for the design of plate girders, focusing on prismatic members with web openings. However, when applying its procedure on the tapered plate girders with web opening, the results were found to be overestimating the web shear capacity by 40% compared to the experimental results. This is due to the guide’s lack of consideration of web slenderness and local buckling which are essential to take into account for accurate prediction of shear capacity of such girders. The maximum nominal shear capacity at web opening in this code can be calculated using equations from Eq. 11 and Eq. 12.

11 $$\:V={V}_{pb}+{V}_{pt}$$

where V_pb_ or V_pt_ is the plastic shear capacity of a tee that can be determined next by Eq. 12.

12 $$\:{V}_{pb}\:or\:{V}_{pt}=\:\frac{{F}_{y}{t}_{w}s}{\sqrt{3}}$$

where s is the depth of tee.

### AISC design guide 25

AISC Design Guide 25 focuses on the design of tapered beams specifically. This guide was closest among the comparison between other codes and researches, which is due the guides significant consideration of tapered girders including its slenderness where it accounts for local buckling. However, when its procedure was applied on the specimens in this study considering the reduction of web cross-sectional area for the opening calculating net area, it was found that it underestimated the shear capacity by about 22% conservation. This is due to the neglection of the enhancement caused by the tapering of web on the shear capacity and the stress redistribution that it will lead to according to Ibrahim et al.^6,7^. Nevertheless, the guide succeeded to predict the shear capacity of specimens S2 and S6, where these specimens had web panel areas relatively smaller than other specimens which reduced the other negative effect occurred by the presence of web opening other than the reduction of the web cross-sectional area. The maximum shear strength of stiffened webs using tension field action can be calculated using equations from Eq. 13 to Eq. 16.

For web panels in which flanges infracting a or b:

13 $$\:{V}_{n}=0.6{F}_{y}{A}_{w}\:for\:\frac{{h}_{avg}}{{t}_{w}}\le\:1.10\sqrt{\frac{{k}_{v}E\:}{{F}_{y}}}$$

14 $$\:{V}_{n}=0.6{F}_{y}{A}_{W}\left({C}_{v}+\frac{1-{C}_{v}}{1.15\left(a/{h}_{min}+\sqrt{1+{\left(a/{h}_{min}\right)}^{2}}\right)}\right)\:for\:\frac{{h}_{avg}}{{t}_{w}}>1.10\sqrt{\frac{{k}_{v}E\:}{{F}_{y}}}$$

where *A*_*w*_ *= h*_*avg*_*t*_*w*_. *h*_*avg*_ is the average tapered web panel height. *h*_*min*_ = the smallest height in the panel. k_v_ is the web plate buckling coefficient and can be determined using Eq. 12.

15 $$\:{k}_{v}=5\:\left(\text{f}\text{o}\text{r}\:\text{w}\text{e}\text{b}\:\text{p}\text{a}\text{n}\text{e}\text{l}\text{s}\:\text{w}\text{i}\text{t}\text{h}\text{o}\text{u}\text{t}\:\text{s}\text{t}\text{i}\text{f}\text{f}\text{e}\text{n}\text{e}\text{r}\text{s}\right)$$

16 $$\:{k}_{v}=5+\frac{5}{{\left(a/{h}_{avg}\right)}^{2}}\left(\text{f}\text{o}\text{r}\:\text{w}\text{e}\text{b}\:\text{p}\text{a}\text{n}\text{e}\text{l}\text{s}\:\text{w}\text{i}\text{t}\text{h}\:\text{s}\text{t}\text{i}\text{f}\text{f}\text{e}\text{n}\text{e}\text{r}\text{s}\right)$$

where a is the clear distance between stiffeners. h_avg_ is the distance between flanges.

Mentioning that the opening in web is taken into consideration as a reduction in the substitution of A_net_ instead of A_w_. where A_net_ is A_w_ reduced by the web opening cross sectional area.

### Eurocode 3

Eurocode 3 provides guidelines for designing of steel plated elements and it includes provisions for members with web openings. It specifies to reduce the web cross-sectional area by the area of opening to calculate the net web cross-sectional area to advance and calculate the web shear capacity. However, when applying its procedure on the specimens, the results were overestimating the shear capacity compared to experimental results by an average of 28%. This indicates that the code doesn’t fully consider the local buckling and stress redistribution caused by the presence of web openings in its methodology. Nevertheless, it predicted the shear capacity of S3, S4 and S7, where these specimens relatively have the smallest web opening diameters compared to others which reduced the influence of the presence of web openings in these specimens. The maximum shear strength of stiffened webs using tension field method can be determined using equations from Eq. 17 to Eq. 20.

17 $$\:V =[(dt_{w} \tau_{bb})+0.9 (g t_{w}\sigma_{bb} sin \Phi )]/\gamma_{M1}$$

where d is web depth mentioning that it was reduced to the net average depth of web panel by the web opening diameter, t_w_ web thickness, τ_bb_ is the initial shear buckling strength, g is the tension field width originated, σ_bb_ is its strength and Φ is its inclination and *γ*_*M1*_ is a factor of safety.

18 $$\:{\tau\:}_{bb}={f}_{yw}/\sqrt{3}\:\text{f}\text{o}\text{r}\:{\stackrel{-}{\lambda\:}}_{w}\le\:0.8$$

19 $$\:{\tau\:}_{bb}=\:\left.\left[1\:-\:0.8\left({\stackrel{-}{\lambda\:}}_{w}-\:0.8\right)\right.\right]\left({f}_{yw}/\sqrt{3}\right)\:\text{f}\text{o}\text{r}\:{0.8<\stackrel{-}{\lambda\:}}_{w}<1.25$$

20 $$\:{\tau\:}_{bb}=\:\left.\left[1/{{\stackrel{-}{\lambda\:}}_{w}}^{2}\right.\right]\left({f}_{yw}/\sqrt{3}\right)\:\text{f}\text{o}\text{r}\:{\stackrel{-}{\lambda\:}}_{w}\ge\:1.25$$

where f_yw_ is the web steel yield stress. $\:{\stackrel{-}{\lambda\:}}_{w}=\frac{d/{t}_{w}}{37.5\epsilon\:\sqrt{{k}_{T}}}$, ε is strain coefficient = (235/fy)^0.5^ (f_y_ is the steel yield stress in N/mm^2^). k_T_ is shear buckling factor = 5.34 (for vertical stiffeners at supports and no intermediate stiffeners).

### Egyptian code of practice (ECP)

The Egyptian Code of Practice provides general guidelines for the design of steel girders. When its procedures were applied to the specimens, the results obtained were very conservative by an average of 58% compared to the experimental results. This is due to the code’s simplified approach, which does not fully address the effect of tapering and presence of web openings. It focuses on prismatic members and assumes uniform stress distributions. The maximum shear strength of stiffened webs can be determined using equations from Eq. 21 to Eq. 26.

21 $$\:V={A}_{w}{q}_{b}$$

where A_w_ is the web cross sectional area while taking the web opening into consideration in the reduction of web cross sectional area by the area of web opening. q_b_ is the allowable shear stress and can be calculated from Eqs. 25 & 26

22 $$\:{q}_{b}={0.35F}_{y}\:\text{f}\text{o}\text{r}\:{\lambda\:}_{q}\le\:0.8$$

23 $$\:{q}_{b}=\:\left(1.5\:-\:0.625{\lambda\:}_{q}\right)\left({0.35F}_{y}\right)\:\text{f}\text{o}\text{r}\:0.8<{\lambda\:}_{q}<1.2$$

24 $$\:{q}_{b}=\:\left(\frac{0.9}{{\lambda\:}_{q}}\right)\left({0.35F}_{y}\right)\:\text{f}\text{o}\text{r}\:{\lambda\:}_{q}\ge\:1.2$$

where F_y_ is the steel yield stress. λ_q_ is the web slenderness parameter $\:=\frac{d/{t}_{w}}{57}\sqrt{\frac{Fy}{{k}_{q}}}$. k_q_ is shear buckling factor shear.

25 $$\:{k}_{q}=4+\frac{5.34}{{\alpha\:}^{2}}\:if\:\alpha\:\le\:1.0$$

26 $$\:{k}_{q}=5.34+\frac{4}{{\alpha\:}^{2}}\:if\:\alpha\:>1.0\:\alpha\:=\frac{\text{s}\text{p}\text{a}\text{c}\text{i}\text{n}\text{g}\:\text{o}\text{f}\:\text{t}\text{r}\text{a}\text{n}\text{s}\text{v}\text{e}\text{r}\text{s}\:\text{s}\text{t}\text{i}\text{f}\text{f}\text{e}\text{n}\text{e}\text{r}\text{s}}{web\:depth}$$

### Serror et al.^2^

The study proposed a methodology for predicting the shear capacity of tapered girders, considering the effects of local buckling, slenderness, and stress redistribution. However, when applying its procedure on the specimens, it overestimated the shear capacity by an average of 45%. This indicated that the approach may not fully capture the actual shear behavior of girders with web openings where their presence significantly reduces the shear capacity. While the study provides valuable insights, its methodology appears to overestimate the effect of certain parameters. This indicates the need for refining the approach considering the presence of web opening. They proposed a shear buckling coefficient for predicting the shear capacity of tapered girders accurately when applying on Eq. 13 from Eurocode 3, The maximum shear capacity from this procedure can be calculated using Eq. 27

27 $$V=K_{n}[(dt_{w} \tau_{bb})+0.9\:(g\:t_{w} \sigma_{bb} sin \Phi )]/1.05$$

where, K_n_ is the nominal shear buckling coefficient, d is web depth, t_w_ web thickness, τ_bb_ is the initial shear buckling strength and g is the tension field width originated, σ_bb_ is its strength and Φ is its inclination.

They proposed the nominal shear buckling coefficient K_n_ that’s function of parameters and coefficients with their values included in their research.

### Ibrahim et al.^6^

The study proposed a methodology for predicting the shear capacity of tapered girders. When applying its methodology on the specimens, which include web openings, the predictions were found to be conservative compared to the experimental results by an average of 40%. This conservation is due to the methodology being developed for girders without openings, where the shear resistance is primarily governed by web slenderness and tapering effects. Hence, the methodology does not fully account for the influence of openings on stress redistribution and shear capacity reduction. While the study provides valuable insights, its direct application to girders with web openings may require further improvements. They proposed shear buckling factor for shear for predicting the shear capacity of tapered girders when applying on Eq. 6 from AISC 360. The maximum shear capacity from this procedure can be calculated using equations from Eq. 28.

28 $$\:{V}_{n}=0.6{F}_{y}{A}_{w}{C}_{vt}$$

where F_y_ is the steel yield stress, A_w_ is the web cross sectional area and C_vt_ is the shear buckling stress to shear stress ratio, it takes web tapering and aspect ratio effects into consideration by using their proposed coefficient of shear buckling k_vtFR_ and correction factor C_r_. They can be calculated using equations from Eq. 29 to Eq. 35.

29 $$\:{k}_{vtFR}={k}_{vtSS}+{\beta\:}_{vt}\left({k}_{vtFF}-{k}_{vtSS}\right)$$

30 $$\:{k}_{vtSS}=5.907-\frac{0.604}{{R}^{2}}+\frac{8.202}{\alpha\:}-\frac{6.748}{\left(\alpha\:R\right)}$$

31 $$\:{k}_{vtFF}=9.775-\frac{0.558}{{R}^{2}}+\frac{13.558}{\alpha\:}-\frac{12.358}{\left(\alpha\:R\right)}$$

32 $$\:{\beta\:}_{vt}=\left({t}_{f}/{t}_{w}\right)-0.64-0.16\:{\left({t}_{f}/{t}_{w}\right)}^{2}\le\:1.00$$

33 $$\:{C}_{r}=0.87-\frac{0.635}{R}-\frac{65.68}{R\left(h/{t}_{w}\right)}$$

34 $$\:{C}_{vt}=1.0\:if\:\:h/{t}_{w\:}\le\:1.10\sqrt{{k}_{vtFR}E/{F}_{y}}$$

35 $$\:{C}_{vt}={C}_{r}\frac{\sqrt{{k}_{vtFR}E/{F}_{y}}}{h/{t}_{w}}\le\:\frac{1}{R}\:if\:\:h/{t}_{w\:}>1.10\sqrt{{k}_{vtFR}E/{F}_{y}\:}$$

### R.I. Shain et al.^14^

This study proposed a methodology for determining the coefficient of elastic local critical buckling. Applying this method by calculating the buckling coefficient by their method and advancing to calculating the shear capacity, it was found that it was conservative by an average of 48% compared to the experimental results. Although they considered the tapering ratio and slenderness ratio, the web opening presence had additional effects that need to be considered in the study to determine the shear capacity of such girders. Their shear buckling coefficient can be calculated from Eq. 36 to 38.

36 $$\:k\:=\:{\gamma\:}_{k}*\:\left(20-\frac{7.78}{{\alpha\:}^{1.34}}+\frac{17.19}{{R}^{0.27}}-\frac{0.186}{\left({{\alpha\:}^{3.97}R}^{1.82}\right)}\right)$$

37 $$\:{\gamma\:}_{k}=\left\{\begin{array}{c}1,\:\:R=1\\\:{{\lambda\:}_{n}}^{0.09},\:\:R\ne\:1\end{array}\right.$$

where k is the shear buckling coefficient, γk is a correction factor and λn is the normalized slenderness ratio.

38 $$\:{\lambda\:}_{n}=\frac{h}{100{t}_{w}}$$

Considering that, the maximum shear capacity (V) was calculated using the rest of AISC procedure in Eq. 5 and to calculate (Cv)in advance using Eq. 6 to 8.

**Fig. 16:**
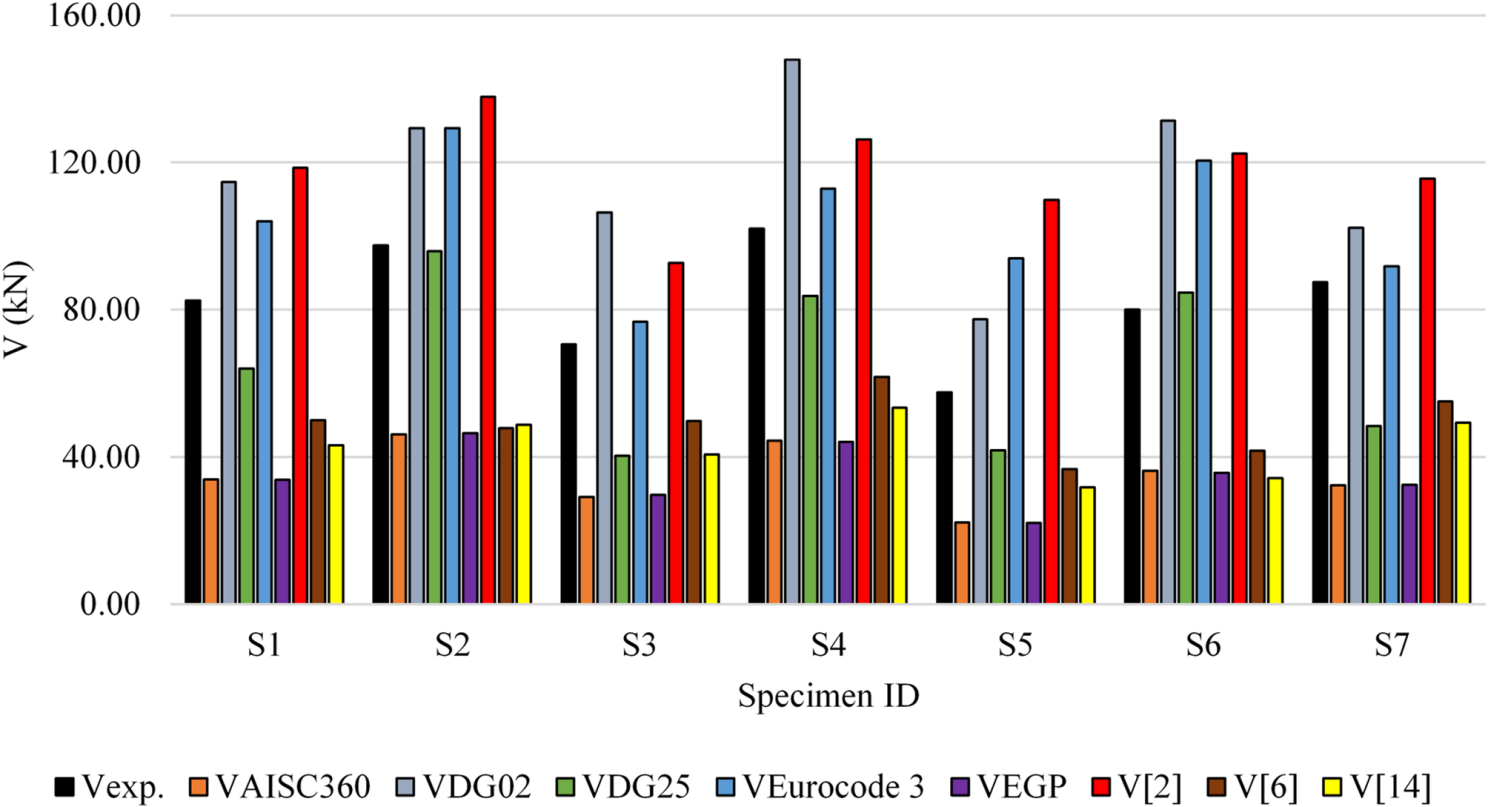
Comparison between results from experimental and specifications results.

Table 4; Fig. 16 show comparison between experimental results of this research and the calculated capacities using previously mentioned design codes and earlier researches.

**Table 4:** Comparison between results from experimental and specifications results.

Specimen	V_exp_.	V_AISC360_	V_DG02_	V_DG25_	V_Eurocode 3_	V_EGP_	V_[2]_	V_[6]_	V_[14]_
ID	kN	kN	kN	kN	kN	kN	kN	kN	kN
S1	82.50	33.96	114.73	63.98	103.99	33.74	118.55	49.99	43.18
S2	97.50	46.11	129.28	95.80	129.38	46.47	137.88	47.85	48.77
S3	70.50	29.09	106.42	40.38	76.69	29.73	92.69	49.81	40.67
S4	102.00	44.41	147.99	83.67	112.92	44.12	126.29	61.76	53.34
S5	57.50	22.21	77.32	41.84	93.94	22.06	109.84	36.76	31.75
S6	80.00	36.29	131.36	84.57	120.45	35.70	122.42	41.76	34.25
S7	87.50	32.34	102.26	48.42	91.78	32.39	115.64	55.02	49.37

## Conclusions

This research presented an experimental study to measure the capacity of slender web tapered plate girder with web opening. Seven samples with different configurations were tested to investigate the impact of web aspect ratio (B/H), opening diameter / average web height (ϕ/h_avg_) and tapering ratio on the web capacity. The recorded results were used to develop a formula to predict the web capacity and to evaluate the accuracies of commonly used design codes.

A thorough investigation of the parameters influencing web capacity revealed:

- Aspect Ratio (B/H): Larger aspect ratios increased panel areas, amplifying buckling vulnerability and reducing web capacity.
- Web Opening Size (D/h_avg_.) Increasing opening sizes linearly reduced the effective shear-resisting area, negatively impacting performance.
- Tapering Ratio (tan θ): Higher tapering ratios improved shear performance by redistributing stresses effectively and increasing critical web depth.

The proposed formula for predicting the web capacity of tapered plate girders with web openings, incorporating key parameters such as the aspect ratio (B/H), web opening size (D/h_avg_.), and tapering ratio (tan θ). The equation was rigorously validated against experimental results, demonstrating a maximum error of just 8%.

In addition, a detailed comparison between the proposed formula, experimental results, and existing specifications highlighted critical gaps in current design methodologies. AISC 360 Specifications overly conservate results, underestimating shear capacity by an average of 58%, due to a lack of provisions for tapered members and simplified web buckling treatment. AISC Design Guide 2 overestimated shear capacity by 40%, as it neglects slenderness and local buckling, crucial for tapered girders with web openings. AISC Design Guide 25 had conservative predictions (22%) caused by simplified assumptions about stress redistribution in tapered configurations. Eurocode 3 overestimated capacity by 28%, reflecting limitations in stress redistribution and slender web buckling considerations. Egyptian Code of Practice had highly conservative (58%) due to its simplified methodology, which is better suited to prismatic members. Serror et al. (2017) overestimated shear capacity (45%) as the method did not account for the reduction in shear resistance due to web openings. Ibrahim et al. (2020) had conservative predictions (40% underestimation) as the method was designed for girders without openings, lacking the adaptability needed for web openings. R.I. Shahin et al. (2023) underestimated shear capacity by (48%) as the presence of web opening caused additional effects that needs to be considered.

It should be noted that the proposed formula is valid within the considered range of each studied parameter and it must be verified beyond these ranges.

For future studies, it is recommended to extend the ranges of the considered parameters and conducting a full numerical parametric study to generate a database large enough to develop a wider range predictive model using ML techniques.

## Data Availability

All generated data are included in Table 2.
